# Emerging antibody–drug conjugates in lung cancer: highlights from the 2026 ASCO Annual Meeting

**DOI:** 10.3389/fonc.2026.1943176

**Published:** 2026-10-06

**Authors:** Xiaolei Wang, Jiayuan Miao, Yuxuan Bao, Wanli Wang, Xin Yan, Mei Wang, Hongchang Shen

**Affiliations:** 1 Department of Oncology, Provincial Hospital Affiliated to Shandong First Medical University, Jinan, Shandong, China; 2 Department of Pharmacy, Shandong Provincial Hospital Affiliated to Shandong First Medical University, Jinan, Shandong, China

**Keywords:** ADCs, immunotherapy combination, non-small cell lung cancer, small-cell lung cancer, targeted therapy

## Abstract

The 2026 American Society of Clinical Oncology (ASCO) Annual Meeting presented substantial new data on antibody–drug conjugates (ADCs) in lung cancer, highlighting the ongoing expansion of ADC-based strategies from later-line treatment toward first-line combination regimens. This focused narrative review summarizes and discusses the efficacy and safety findings of ADCs targeting TROP2, HER2, Nectin-4, PD-L1, c-Met, B7-H3, DLL3, SEZ6, and other emerging targets in non-small cell lung cancer (NSCLC) and small cell lung cancer (SCLC), with particular attention to emerging ADC plus immune checkpoint inhibitor combinations in the first-line setting. Taken together, these findings highlight an expanding ADC landscape in lung cancer. However, many of the emerging strategies remain supported primarily by preliminary conference-reported or early-phase data and therefore require confirmation in mature peer-reviewed studies and adequately powered randomized clinical trials.

## 1 Advances in ADC drugs for non-small cell lung cancer

The structural characteristics of the ADCs discussed in this review are summarized in **Table 1**.

**Table 1:** Characteristics of ADCs for the treatment of lung cancer.

ADCs	Target	mAb	Linker	Payload	DAR
Sac-TMT	TROP2	Sacituzumab	MSP	KL610023	7.4
FZ-AD004	TROP2	Anti-TROP2 mAb	CL	Dxd	3.8
HLX43	PD-L1	HLX20	Cleavable and TME-activatable tripeptide linker	C24	8
TQB6411	EGFR and c-Met	EGFR × c-Met bsAb	Mc-GGFG linker	DXd	NR
BL-M14D1	DLL3	Anti-DLL3 mAb	IMP042	Ed-04	8
DXC006	CD56	Anti-CD56 mAb	cleavable peptidyl bis-linker	CPT113	4
SYS6043	B7-H3	Anti-B7-H3 mAb	CL	Topoisomerase I inhibitor	6
SKB500	B7-H3	Anti-B7-H3 mAb	cleavable pyrimidine-tripeptide linker	Topoisomerase I inhibitor	≈8
ABBV-706	SEZ6	Anti-SEZ6 mAb	CL	topoisomerase I inhibitor	6
SV	IB6	Sigotatuzumab	Mc-vc	MMAE	4
SHR1826	c-Met	Humanized IgG2 monoclonal anti-c-Met mAb	Cleavable peptide-based linker	Topoisomerase I inhibitor	NR
BB-1701	HER2	Trastuzumab	Mc-PEG2-Val-Cit-PABC	Eribulin	NR
SHR-A2102	Nectin-4	Anti-Nectin-4 mAb	CL	Topoisomerase I inhibitor	NR
ABBV-400	c-Met	telisotuzumab	CL	Adizutecan	Optimized DAR
T-DXd	HER2	Trastuzumab	GGFG linker	DXd	8
TAK-188	CCR8+	Anti-CCR8 mAb	CL	amanitin	NR
DS3610	EGFR	NR	CL	CDN STING agonist	NR
BNT326/YL202	HER3	Anti-HER3 IgG1 mAb	tripeptide linker	Topoisomerase I inhibitor	≈8
ZL-1310	DLL3	anti-DLL3 mAb	Protease-CL	Topoisomerase I inhibitor	8

ADCs, Antibody-drug conjugates; mAb, monoclonal antibody; DAR, Drug-to-antibody ratio; MSP, methylsufonyl-pyrimidine; DXd, deruxtecan payload; CL, cleavable linker; MMAE, MonomethylAuristatin E; MC-VC, Maleimidocaproyl-Valine-Citrulline; Ed-04, Edotecarin-04; CDN, Cyclic Dinucleotide; Mc-PEG2-Val-Cit-PABC, Maleimidocaproyl-polyethylene glycol 2-valine-citrullinep-aminobenzyloxycarbonyl; SV, sigvotatug vedotin; NR, Not Reach; Sac-TMT, Sacituzumab Tirumotecan; IB6, Integrin beta-6.

### 1.1 TROP2-targeting ADCs: emerging first-line combination strategies

TROP2 has emerged as an important therapeutic target across solid tumors. In the randomized phase III OptiTROP-Lung05 trial, 413 patients with PD-L1-positive advanced NSCLC were assigned to sacituzumab tirumotecan (Sac-TMT) plus pembrolizumab (n = 208) or pembrolizumab alone (n = 205) as first-line treatment. At a median follow-up of 10.5 months, the BICR-assessed median PFS had not been reached with Sac-TMT plus pembrolizumab versus 5.7 months with pembrolizumab alone (HR, 0.35; 95% CI, 0.26–0.47). The ORR was 70.2% versus 42.0%, respectively. OS data remained immature, although a favorable trend was observed with Sac-TMT plus pembrolizumab (HR, 0.55; 95% CI, 0.36–0.85). These findings provide randomized phase III evidence supporting the clinical activity of Sac-TMT plus pembrolizumab in first-line PD-L1-positive advanced NSCLC. Grade ≥3 TEAEs occurred in 55.3% of patients receiving Sac-TMT plus pembrolizumab versus 31.4% receiving pembrolizumab alone, with decreased neutrophil count (17.3%), anemia (9.1%), and stomatitis (5.3%) among the most common grade ≥3 TEAEs in the combination arm. TEAEs led to discontinuation of Sac-TMT and pembrolizumab in 3.8% and 5.3% of patients in the combination arm, respectively, versus 4.9% for pembrolizumab in the control arm. However, the relatively short follow-up and immature survival data warrant continued observation before the durability of benefit and long-term benefit–risk profile can be fully established (1).

FZ-AD004 is a TROP2-targeting ADC armed with the topoisomerase I inhibitor DXd. In a phase I dose-escalation trial, 12 efficacy-evaluable patients receiving FZ-AD004 at doses ≥8.0 mg/kg achieved an ORR of 50.0% and a DCR of 83.3%. Tumor regression was observed across several molecular subgroups, including KRAS G12C-mutant, EGFR-mutant, and oncogene-negative tumors. However, given the small number of efficacy-evaluable patients, these findings should be considered preliminary and are insufficient to establish that the activity of FZ-AD004 is independent of underlying genomic alterations. The principal reported toxicities included stomatitis, decreased lymphocyte count, and nausea. Given the early-phase design and limited sample size, the long-term tolerability of FZ-AD004 remains to be established (2).

### 1.2 HER2-targeting ADCs: emerging evidence in patients with brain metastases

Trastuzumab deruxtecan (T-DXd) has demonstrated clinically meaningful antitumor activity in HER2-mutant NSCLC. In a *post hoc* subgroup analysis of DESTINY-Lung05, 72 Chinese patients with metastatic HER2-mutant NSCLC were evaluated. Among 30 patients with baseline brain metastases, the systemic confirmed objective response rate (ORR) was 46.7%, the disease control rate (DCR) was 90.0%, and the median duration of response (DOR) was 7.1 months. Among 42 patients without baseline brain metastases, the systemic ORR was 64.3%, the DCR was 92.9%, and the median DOR was 14.0 months. Importantly, among 10 patients with measurable CNS disease, the CNS-ORR was 40.0% and the CNS-DCR was 100%, with CNS-specific progression outcomes evaluated separately. These findings suggest intracranial activity of T-DXd in HER2-mutant NSCLC; however, the small measurable-CNS subgroup and *post hoc* nature of the analysis limit the precision and generalizability of these estimates. ILD/pneumonitis remains an important safety concern with T-DXd, and the available CNS subgroup data do not establish a distinct safety profile (3).

BB-1701 is a HER2-directed ADC carrying an eribulin payload. In an ongoing phase II study of BB-1701 plus sintilimab in HER2-low breast cancer or HER2-mutant NSCLC, 12 patients were treated, including 7 with NSCLC. Among the patients with NSCLC, two achieved partial responses and two had stable disease, yielding a DCR of 57.1%. Three grade 3 TEAEs were reported in the overall study population: γ-glutamyltransferase increased, pneumonia, and open globe injury. These preliminary findings support further investigation of the combination, but the small NSCLC subgroup and limited safety population preclude definitive conclusions regarding efficacy, predictive biomarkers, or tolerability (4).

### 1.3 Nectin-4-targeting ADCs: a new contender among emerging targets

SHR-A2102 is a nectin-4-directed ADC carrying a topoisomerase I inhibitor payload. In a multicenter, open-label phase 1b/II trial evaluating SHR-A2102 plus adebrelimab as first-line therapy for locally advanced or metastatic NSCLC, 59 efficacy-evaluable patients achieved an ORR of 74.6%, a DCR of 96.6%, a median PFS of 9.9 months, and a median duration of response (DOR) that was not yet reached. Grade ≥3 TRAEs occurred in 36/67 patients (53.7%), mainly involving hematologic toxicity (5). Collectively, these preliminary findings provide clinical support for further investigation of Nectin-4 as a therapeutic target and demonstrate encouraging antitumor activity of SHR-A2102 plus adebrelimab in untreated advanced NSCLC. Nevertheless, the single-arm early-phase design and limited follow-up preclude definitive conclusions regarding comparative efficacy or its role in the first-line treatment landscape.

### 1.4 PD-L1-targeting ADCs: fusion of immunotherapy and chemotherapy

HLX43 is a novel PD-L1–targeted ADC comprising a fully human IgG1 anti-PD-L1 backbone, a tumor microenvironment–activatable tripeptide linker, a C24 topoisomerase I inhibitor payload, and a uniform drug-to-antibody ratio of 8, enabling dual antitumor activity via direct cytotoxicity and PD-L1 blockade–mediated immunomodulation. Pooled phase I/II efficacy data from 205 heavily pretreated advanced NSCLC patients were presented at the 2026 ASCO Annual Meeting. In the 2.0 mg/kg squamous cohort (n = 33), the confirmed ORR was 33.3% with a median PFS of 6.34 months, whereas the corresponding values were 46.7% and 6.90 months in patients with prior docetaxel failure (n = 15). In the 2.5 mg/kg non-squamous cohort, EGFR wild-type (n=19) and EGFR-mutant (n=16) patients achieved ORRs of 36.8% and 37.5%, with median PFS of 6.67 and 5.55 months, respectively. Responses were observed in both reported PD-L1 subgroups, with ORRs of 37.9% and 33.3%, respectively. However, these subgroup observations do not establish independence from PD-L1 expression and are insufficient to define HLX43 as a biomarker-agnostic treatment strategy. Prospective validation in larger biomarker-defined cohorts is required. Grade ≥3 TRAEs occurred in 42.9% of patients, predominantly involving hematologic toxicity, while TRAEs led to treatment discontinuation in 8.3%. Longer follow-up is required to further characterize tolerability (6).

### 1.5 Dual-targeting ADCs: dual inhibition of EGFR and c-Met

TQB6411 is a first-in-class bispecific antibody–drug conjugate (ADC) co-targeting EGFR and c-Met, engineered with a 2:1 c-Met/EGFR binding affinity ratio to potentiate cytotoxicity against c-Met–overexpressing tumors while mitigating EGFR-driven cutaneous toxicities. In a phase I dose-escalation trial (data cutoff: January 13, 2026), no dose-limiting toxicities were observed up to 6.6 mg/kg. Among seven efficacy-evaluable patients with NSCLC treated at 4 mg/kg, the ORR was 42.9% and the DCR was 100%. These preliminary findings support further investigation of the biased-affinity design. However, the very small efficacy-evaluable cohort precludes definitive conclusions regarding antitumor efficacy or a reduction in EGFR-related toxicity. The absence of observed DLTs does not establish overall safety, and longer follow-up is required to characterize tolerability (7).

### 1.6 Other emerging targets

SHR-1826, a c-Met-targeted ADC, demonstrated consistent efficacy across molecular subsets of advanced NSCLC. Updated analyses in EGFR-mutant lung adenocarcinoma (n = 36) showed an ORR of 41.7%, DCR of 94.4%, mPFS of 9.8 months, and mDOR of 14.1 months (8). SYS6043, a B7-H3-targeted topoisomerase I inhibitor ADC, achieved a confirmed ORR of 46.3% in lung cancer, rising to 66.7% in SCLC, with an mDOR of 6.9 months (9). In treatment-naïve advanced NSCLC, the IB6-directed ADC in combination with pembrolizumab achieved confirmed ORRs of 50–61% (DCR: 90–94%) and 44–63% (DCR: 89–95%) by histologic subtype, with a median duration of response of 8.1 months (not reached in the TPS <1% and squamous subgroups) (10). These findings support the ongoing phase III SigVie-003 trial (11). Although these findings demonstrate encouraging early antitumor signals across several emerging targets, most were derived from non-randomized early-phase cohorts. Cross-study comparisons should therefore be avoided, and the durability and comparative clinical value of these agents remain to be established in larger prospective studies.

## 2 Advances in ADCs for small cell lung cancer

SCLC is a highly aggressive neuroendocrine tumor with historically limited therapeutic options. At ASCO 2026, several ADCs targeting distinct antigens showed preliminary antitumor activity in SCLC.

### 2.1 DLL3-targeting ADCs: precision attack on SCLC

BL-M14D1 is a DLL3-targeted ADC carrying the topoisomerase I inhibitor Ed-04. In the phase I study, 127 patients were enrolled, including 87 with SCLC. Among 83 efficacy-evaluable patients with SCLC treated at 4.0 or 4.5 mg/kg, the ORR was 71.1% (95% CI, 60.1–80.5), the confirmed ORR was 57.8% (95% CI, 46.5–68.6), the DCR was 94.0% (95% CI, 86.5–98.0), and the median PFS was 7.2 months (95% CI, 5.6–12.7). These early-phase findings support further clinical investigation of BL-M14D1 in SCLC but require confirmation in randomized studies (12).

### 2.2 SEZ6-targeting ADCs

In a phase I trial of the SEZ6-targeted ADC ABBV-706 (N = 135, including 11 combination-therapy patients), the monotherapy cohort (N = 124) achieved an ORR of 52%, with a median PFS of 5.4 months and a median OS of 11.3 months. An exploratory combination with budigalimab (anti-PD-1, n=11) yielded an ORR of 54.5% and a median PFS of 8.1 months.

These preliminary findings support further evaluation of ABBV-706 as monotherapy and in combination with immunotherapy in SCLC; however, the small exploratory combination cohort precludes reliable comparison between treatment strategies (13).

### 2.3 B7-H3-targeting ADCs

SKB500, a novel B7-H3–directed ADC, showed encouraging preliminary antitumor activity in a first-in-human phase I dose-escalation study. In 40 patients with relapsed/refractory SCLC, SKB500 achieved an ORR of 65.0%, a median PFS of 7.2 months, and a median DOR of 5.8 months. At the 12 mg/kg dose level, a low permanent discontinuation rate and no treatment-emergent deaths were reported (14). Collectively, these findings provide a rationale for continued clinical development of SKB500 in SCLC.

### 2.4 CD56-targeting ADCs

DXC006 is a CD56-targeted ADC carrying a topoisomerase I inhibitor payload. In the phase I study, SCLC-specific antitumor activity was observed in the efficacy-evaluable subgroup, with an ORR of 69.4% (95% CI, 53.1–82.0) and a DCR of 91.7% (95% CI, 78.2–97.1). Among patients treated in the second-line setting, the ORR was 85.7% (95% CI, 60.0–96.0) and the DCR was 100%. These findings suggest preliminary activity in previously treated SCLC, although confirmation in larger disease-specific cohorts is required (15). 

## 3 Ongoing phase III trials reported at ASCO 2026

The meeting featured a broad portfolio of ADC trials across NSCLC and SCLC (**Table 2**). In addition, key ongoing studies include the phase III SigVie-003 trial of the IB6-targeting ADC sigvotatug vedotin (SV) plus pembrolizumab in first-line NSCLC (11); the randomized phase II SEZanne study evaluating ABBV-706 plus atezolizumab versus standard-of-care therapy in patients with previously untreated extensive-stage small cell lung cancer (ES-SCLC) (16); and the phase III DLLEVATE trial of the DLL3-targeting ADC ZL-1310 in relapsed SCLC (17). Telisotuzumab adizutecan (ABBV-400; Temab-A), a c-Met–directed ADC, is being evaluated across multiple phase II/III trials in EGFR-mutated NSCLC, including combination regimens with osimertinib versus standard-of-care chemotherapy (18, 19). Earlier-phase programs encompass TAK-188 (CCR8-targeted, α-amanitin payload) (20), DS3610a (EGFR-targeted, STING agonist payload) (21), and BNT326/YL202 (HER3-targeted, with or without pumitamig) (22), all of which are being evaluated in NSCLC. Comprehensive details for each trial are provided in **Table 3**. These ongoing studies are expected to clarify whether the preliminary efficacy signals observed in early-phase trials translate into clinically meaningful benefit in larger, controlled populations.

**Table 2:** Outcomes of ADCs for the treatment of lung cancer from ASCO 2026.

Drug	Indication	Agents	Pts	Phase	Treatment line	ORR (%)	OS	PFS (months)	DOR (months)	DCR (%)	Grade ≥3 AEs/SAEs	DLTs	Discontinuation/dose modification	Key AESIs	References	NCT
Sac-TMT	NSCLC	Sac-TMT+Pembro vs Pembro	413 (208 vs 205)	III	1L	70.2 vs 42.0	Immature; HR 0.55 (95% CI, 0.36–0.85)	- vs 5.7; HR 0.35 (95% CI, 0.26–0.47)	–	–	TEAEs: 55.3% vs 31.4%, SAEs: -	–	Sac-TMT 3.8%; pembro 5.3% vs 4.9% (combination vs control); dose modification -	Grade ≥3: neutrophil count decreased 17.3%; anemia 9.1%; stomatitis 5.3%	(1)	NCT06448312
FZ-AD004	NSCLC, SCLC	FZ-AD004 dose-escalation	22	I	≥1L	50 (Among 12 efficacy-evaluable pts at doses ≥8.0 mg/kg)	–	–	–	83.3	Grade ≥3 TRAEs: 36.4%; SAEs: 22.7%; serious TRAEs: 13.6%	No DLTs observed across all dose levels; MTD not reached.	TRAEs leading to dose reduction: 4/22 (18.2%); treatment discontinuation: 0.	Grade ≥3 stomatitis: 13.6%; decreased lymphocyte count: 9.1%.	(2)	NCT05914545
T-DXd	NSCLC (HER2m)	T-DXd dose-escalation	72 total; BL CNS mets n=30; measurable CNS disease n=10	II	≥1L	BL CNS mets (n=30): 46.7 No BL CNS mets (n=42): 64.3 CNS-ORR: 40.0 (measurable CNS disease)	16.6 24.0	8.0 13.0 CNS-PFS: 7.8 (BL CNS mets)	7.1 14.0 CNS-DOR: NE	90.0 92.9 CNS-DCR: 100	Drug-related grade ≥3 AEs: 60.0% (18/30) with baseline CNS metastases vs 52.4% (22/42) without CNS metastases; SAEs: -	–	–	Specific AESI rates –	(3)	NCT05246514
BB-1701	HER2-low breast cancer or HER2-mutant NSCLC	BB-1701+Sintilimab	12 total (7 NSCLC)	II	≥1L	PD-L1 low (n=8): 25 PD-L1 high (n=2): 50	- -	- -	- -	62.5 100	Grade 3 TEAEs: 3 events (overall N = 12); SAE: 1 event	–	–	Peripheral neuropathy; hepatic and hematologic toxicities; ocular event.	(4)	CTR20241422
SHR-A2102	NSCLC	SHR-A2102+Adebrelimab	67	Ib/II	1L	Total (n = 59): 74.6	–	9.9	NR	96.6	Grade ≥3 TRAEs: 36/67 (53.7%); SAEs: -	–	–	No reported; prominent toxicities included myelosuppression (neutropenia, leukopenia, anemia), alopecia, and gastrointestinal/hepatic AEs.	(5)	NCT06512051
HLX43	NSCLC	HLX43 dose-escalation	205	I/II	≥1L	Squamous 2.0 mg/kg (n=33): 33.3 EGFR wild-type NSQ (n=19): 36.8 EGFR mutant NSQ (n=16): 37.5	- - -	6.34 6.67 5.55	- - -	81.8 94.7 87.5	Grade ≥3 TRAEs: 42.9%; mainly hematologic; SAEs: -	–	TRAE-related discontinuation: 8.3%; dose modification No reported.	No reported; hematologic toxicities predominated.	(6)	NCT06115642 NCT06907615
TQB6411	NSCLC	TQB6411 dose-escalation	21 NSCLC (7 efficacy-evaluable at 4 mg/kg)	I	≥1L	42.9 (Among 7 efficacy-evaluable pts)	–	–	–	100	Grade 3 TRAEs: 6 events (N = 23); SAEs: -	None observed up to 6.6 mg/kg.	–	No ILD; infusion reactions 50.0% (38.9% at ≥4 mg/kg after prophylaxis modification).	(7)	NCT07043751
SHR-1826	Lung cancer	SHR-1826 dose-escalation	126	I	≥1L	41.7 (Among 36 efficacy-evaluable pts)	NR	9.8	14.1	94.4	Grade ≥3 TRAEs: 129/195 (66.2%); AEs: -	–	TRAE-related discontinuation: 8/195 (4.1%); dose modification No reported.	ILD: 4/126 (3.1%), including grade ≥3 in 2/126 (1.6%).	(8)	NCT06094556
SYS6043	Lung cancer	SYS6043 dose-escalation	168	I/II	≥1L	46.3 (Among 134 efficacy-evaluable pts)	–	–	6.9	82.8	Grade ≥3 TRAEs: 28.5%; SAEs: -	Grade 3 gastrointestinal disease and grade 4 febrile neutropenia at 10.0 mg/kg Q3W.	–	No reported; prominent toxicities included anemia, neutropenia, nausea, and decreased appetite.	(9)	ChiCTR2400094683
SV	NSCLC	SV+P	37	I	1L	PD-L1 TPS < 1% (n=10): 50 PD-L1 TPS ≥1%(n=18): 61 NSQ(n=19): 63 SQ(n=9): 44	- - - -	- - - -	NR 8.1 8.1 NR	90 94 95 89	Grade ≥3 TEAEs: 59%; treatment-related grade ≥3 TEAEs: 44%; SAEs: -	–	–	No reported; common TEAEs included alopecia, decreased appetite, fatigue, and nausea.	(10)	NCT04389632
BL-M14D1	SCLC, NEC	BL-M14D1 dose-escalation	127 total; 83 efficacy-evaluable SCLC	I	≥1L	71.1	–	7.2	–	94.0	Grade ≥3 TRAEs: 74.0%; SAEs: -	–	TRAE-related discontinuation 1.6%	Grade 3 ILD: 1 case; treatment-related death: 1 case.	(12)	NCT06505824
ABBV-706	SCLC	ABBV-706 monotherapy (124) ABBV 706+budigalimab (11)	135	I	≥ 2L	52 54.5	11.3 NR	5.4 8.1	5.3(n=65) 6.7(n=6)	91.1 81.8	Grade ≥3 TRAEs: 75/124 (60.5%); SAEs: -	–	TRAE-related discontinuation: 10/124 (8.1%); dose interruption 44.4%, reduction 27.4%.	Adjudicated ILD: 7/124 (5.6%) with monotherapy; 0/11 with budigalimab. Treatment-related deaths: 3/124 (2.4%).	(13)	NCT05599984
SKB500	SCLC	SKB500 dose escalation	40	I	≥1L	65.0	–	7.2	5.8	95.0	Grade ≥3 TRAEs: 16/97 (16.5%); SAEs: -	None observed during dose escalation (2–18 mg/kg).	No TRAE-related discontinuation or death; dose modification no reported.	Pneumonitis: 2/97 (2.1%), both grade 1.	(14)	NCT06736327 NCT04389632
DXC006	SCLC, NSCLC, NET	DXC006 dose-escalation	72 total; 36 efficacy-evaluable SCLC	I	≥1L	SCLC 69.4; 2L SCLC 85.7	–	–	–	SCLC 91.7; 2L SCLC 100	TRAEs: 27.8% overall; SAEs: -	None observed; MTD not reached	interruption/discontinuation: 23.6%/1.4%	No ILD observed; sensory disturbance 33.3%, all grade 1–2	(15)	NCT06224855

For OptiTROP-Lung05, comparative values are presented as Sac-TMT + pembrolizumab versus pembrolizumab alone unless otherwise specified. ADCs, Antibody-drug conjugates; DOR, Duration of response; HER2, Human epidermal growth factor receptor 2; ORR, Objective response rate; DCR, Disease Control Rate; OS, Overall survival; Pembro, Pembrolizumab; PFS, Progression-free survival; Pts, Patients; SG, Sacituzumab govitecan; TRAEs, Treatment-related adverse events; T-DXd, Trastuzumab Deruxtecan,– not reported; NR, not reached; NE, not assessed or not estimable; “Not reported” indicates that the endpoint was not provided in the source report and should not be interpreted as absence of the event. BL CNS mets, Baseline Central Nervous System metastases; NSQ, Non-Squamous; SQ, Squamous; NCT, National Clinical Trial; CTR, China Clinical Trial Registration; ChiCTR, Chinese Clinical Trial Registry; SCLC, Small Cell Lung Cancer; NSCLC, Non-Small Cell Lung Cancer; SOC, Standard of Care; SV + P, sigvotatug vedotin＋Pembrolizumab; Sac-TMT + Pembro, Sacituzumab Tirumotecan + Pembrolizumab; HR, Hazard Ratio; NET, Neuroendocrine Tumor; NEC, Neuroendocrine Carcinoma; HER2m, HER2 mutation; TPS, Tumor Proportion Score; AE, adverse event; AESI, adverse event of special interest; DLT, dose-limiting toxicity; SAE, serious adverse event; TEAE, treatment-emergent adverse event; TRAE, treatment-related adverse event. Safety outcomes are reported according to the definitions and analysis populations used in the primary sources.

**Table 3:** Ongoing clinical trials of ADCs in lung cancer.

Drug	Target	Eligibility biomarker	Experimental regimen	Comparator	Phase	Treatment line	Primary endpoint	Planned N	Recruitment status	References	NCT
SV	Integrin beta-6 (IB6)	Locally advanced, unresectable, or metastatic NSCLC; PD-L1 TPS ≥50%; for nonsquamous NSCLC, EGFR/ALK/ROS1 negative and no known actionable genomic alterations with approved 1L therapy	SV 1.8 mg/kg AiBW IV on Days 1, 15, and 29 + pembro 400 mg IV on Day 1, Q6W	Pembro 400 mg IV Q6W	III	1L	OS and BICR-assessed PFS per RECIST v1.1	714	Recruiting	(11)	NCT06758401
ABBV-706	SEZ6	ES-SCLC; ECOG PS 0–1; measurable disease per RECIST v1.1; candidates for platinum-based chemotherapy	ABBV-706 (Dose A or B) + atezolizumab	SOC: carboplatin + etoposide + atezolizumab, with optional lurbinectedin maintenance where applicable	II	1L	Safety/tolerability, dose optimization, and PFS	180	Recruiting	(16)	NCT07155174
ZL-1310	DLL3	ES-SCLC progressing after platinum-based 1L therapy or after tarlatamab as 2L therapy; stable or asymptomatic brain metastases eligible	ZL-1310 IV Q3W until disease progression or unacceptable toxicity	Investigator’s choice: topotecan, lurbinectedin, or amrubicin	III	≥2L	Confirmed ORR by BICR and OS	480	Recruiting	(17)	NCT07218146
ABBV-400 (Temab-A)	c-Met	Locally advanced or metastatic nonsquamous NSCLC with documented EGFR mutation; measurable disease per RECIST v1.1; no prior EGFR TKI treatment in the metastatic setting	Phase II: ABBV-400 1.6, 2.0, or 2.4 mg/kg IV Q3W + osimertinib 80 mg once daily; Phase III: ABBV-400 + osimertinib at the RP3D	SOC: osimertinib + platinum/pemetrexed chemotherapy	II/III	1L	Phase II: safety/tolerability, dose optimization, and BICR-assessed ORR; Phase III: efficacy including BICR-assessed PFS	854	Recruiting	(18)	NCT07005102
ABBV-400 (Temab-A)	c-Met	Locally advanced/metastatic nonsquamous NSCLC with EGFR Ex19del or L858R after progression on 1 prior 3rd-generation EGFR TKI; tumor tissue required for c-Met IHC	Ph2: two doses of telisotuzumab adizutecan monotherapy or + osimertinib; Ph3: RP3D telisotuzumab adizutecan vs SOC	Ph2: dose-level comparison; Ph3: investigator’s-choice platinum-doublet chemotherapy or amivantamab + chemotherapy	II/III	≥2L (after 1 prior 3rd-generation EGFR TKI)	Ph2: BICR-assessed ORR; Ph3: BICR-assessed PFS	490	Recruiting	(19)	NCT07155187
TAK-188	CCR8	Advanced solid tumors with high CCR8 expression on intratumoral Tregs; NSCLC included among expansion cohorts; progressed on/intolerant to standard therapies	TAK-188 IV on Days 1, 8, and 15 of each 21-day cycle during dose escalation, followed by expansion at the RDE/RP2D	None (single-arm dose-escalation study)	I/II	≥2L (after progression on or intolerance to standard therapies)	Ph1: safety/tolerability and RP2D determination; Ph2: preliminary efficacy	223	Recruiting	(20)	NCT07205718
DS3610a	EGFR	Advanced/metastatic solid tumors, including NSCLC; relapsed/refractory to or intolerant of SOC; ECOG PS 0–2; measurable disease per RECIST v1.1; pretreatment or archival tumor tissue required	DS3610a at escalating doses	None (single-arm dose-escalation study)	I	≥2L	Safety/tolerability; determination of recommended dose(s) for expansion (RDE); DLTs and TEAEs	70	Recruiting	(21)	NCT07159126
BNT326/YL202	HER3	Advanced NSCLC; AGA-negative NSCLC or EGFR-mutant NSCLC in specified expansion cohorts	BNT326/YL202 monotherapy and BNT326/YL202 + pumitamig; randomized dose optimization in selected cohorts	No SOC comparator; randomized dose-level comparison in selected cohorts	Ib/II	2L+ for the NSCLC cohorts	Safety/tolerability, ORR, dose optimization, and PK	1438	Recruiting	(22)	NCT07070232

SOC, Standard of Care; NSCLC, Non-Small Cell Lung Cancer; SCLC, Small Cell Lung Cancer; ES-SCLC, Extensive-Stage Small Cell Lung Cancer; SV + P, Sigvotatug vedotin＋Pembrolizumab; ICT, Investigator’s Choice Therapy; osi, Osimertinib; Temab-A, Telisotuzumab Adazutecan; NCT, National Clinical Trial; Recruitment status was obtained from Clinical Trials.gov at the manuscript data cutoff.

## 4 Comparative safety considerations of emerging ADCs

Safety is an important consideration as ADCs move toward earlier treatment lines and combinations with immune checkpoint inhibitors. Safety profiles may differ according to target antigen, antibody and linker characteristics, payload class, dosing schedule, and combination regimen; therefore, findings should be interpreted in the context of each agent and study rather than generalized across ADCs. Clinically relevant toxicities include hematologic adverse events, stomatitis, ILD/pneumonitis, peripheral neuropathy, and ocular toxicity, although their incidence and clinical relevance vary and are not consistently reported. Hematologic toxicity and stomatitis warrant particular attention with topoisomerase I inhibitor–containing ADCs, while peripheral neuropathy may be relevant to microtubule-disrupting payloads. ILD/pneumonitis remains an important adverse event of special interest for selected ADCs, particularly when combined with immunotherapy.

Grade ≥3 adverse events, DLTs, dose interruptions or reductions, permanent treatment discontinuations, and treatment-related deaths are important measures of tolerability and the feasibility of prolonged treatment. However, these outcomes were not uniformly reported across the conference studies. Differences in safety definitions, analysis populations, follow-up duration, and treatment exposure further limit direct cross-trial comparisons. Accordingly, unreported events should not be assumed to be absent, and longer follow-up and more complete safety reporting are needed to establish the long-term benefit–risk profiles of emerging ADC-based strategies.

## 5 Summary and future perspectives

The 2026 ASCO Annual Meeting highlighted the continued expansion of ADCs in lung cancer across multiple targets and treatment settings. The randomized phase III OptiTROP-Lung05 trial provided the most mature evidence for an ADC–immunotherapy combination in first-line PD-L1-positive advanced NSCLC, whereas most other strategies remain supported by early-phase, non-randomized studies with limited sample sizes and immature survival data. These findings therefore require cautious interpretation and further randomized validation.

Key challenges for future development include defining predictive biomarkers, accounting for spatial and temporal heterogeneity in target expression, and understanding resistance mechanisms such as antigen loss and payload-related cross-resistance. Optimal sequencing after prior ADC exposure also remains uncertain, particularly when different ADCs share similar payload classes. CNS activity should be evaluated using dedicated intracranial endpoints, especially in patients with active brain or leptomeningeal metastases.

ADC–immunotherapy combinations are biologically promising, but their clinical benefit must be balanced against overlapping toxicities, including hematologic adverse events, stomatitis, and ILD/pneumonitis. Future progress will depend on biomarker-guided patient selection, rational treatment sequencing, rigorous CNS assessment, and prospective validation of efficacy and safety in randomized trials.

## 6 Limitations

Several limitations should be considered when interpreting the evidence summarized in this review. First, a substantial proportion of the studies discussed were reported as conference abstracts at the 2026 ASCO Annual Meeting and, at the time of this review, had not yet been published as full peer-reviewed articles. Conference abstracts generally provide less comprehensive information regarding study methodology, patient characteristics, efficacy endpoints, and safety outcomes than full publications, and the reported results may change with longer follow-up or subsequent data updates. Second, many of the emerging ADC studies were phase I, phase I/II, or single-arm phase II trials characterized by relatively small and selected patient populations, lack of randomized comparators, and limited follow-up. Consequently, response rates observed in these studies should be interpreted cautiously and cannot by themselves establish comparative efficacy or clinical benefit. In addition, survival outcomes, including duration of response, progression-free survival, and overall survival, remain immature or incompletely reported for several agents. Safety reporting was also heterogeneous across studies, with detailed information on grade ≥3 adverse events, dose modifications, treatment discontinuations, treatment-related deaths, and adverse events of special interest not consistently available. Finally, differences in study populations, treatment lines, biomarker selection, dosing schedules, follow-up duration, and endpoint definitions limit direct cross-trial comparisons. Therefore, findings derived from early-phase or abstract-only reports should be considered preliminary and hypothesis-generating until confirmed by mature peer-reviewed data and, where appropriate, adequately powered randomized clinical trials. And safety reporting was heterogeneous across studies, with detailed information on grade ≥3 adverse events, DLTs, dose modifications, treatment discontinuations, treatment-related deaths, and adverse events of special interest not consistently available. These limitations, together with differences in safety analysis populations and treatment exposure, restrict direct cross-trial comparisons and preclude definitive conclusions regarding the relative tolerability of emerging ADCs.
